# Post-Burn Psychosocial Outcomes in Pediatric Minority Patients in the United States: An Observational Cohort Burn Model System Study

**DOI:** 10.3390/ebj4020015

**Published:** 2023-04-03

**Authors:** Paul Won, Li Ding, Kara McMullen, Haig A. Yenikomshian

**Affiliations:** 1 Keck School of Medicine, University of Southern California, Los Angeles, CA 90033, USA; 2 Department of Population and Public Health Science, Keck School of Medicine, University of Southern California, Los Angeles, CA 90089, USA; 3 Department of Rehabilitation Medicine, University of Washington, Seattle, WA 98195-2100, USA; 4 Division of Plastic Surgery, University of Southern California, Los Angeles, CA 90033, USA

**Keywords:** burns, psychosocial, pediatric, outcomes, burn model system

## Abstract

Racial and ethnic minority burn patients face barriers to longitudinal psychosocial support after injury. Studies utilizing the Burn Model System (BMS) National Database report adult minority patients experience worse psychosocial outcomes in domains such as body image during burn recovery. No study to date has investigated disparities in psychosocial outcomes by racial or ethnic category in the pediatric population using the BMS database. This observational cohort study addresses this gap and examines seven psychosocial outcomes (levels of anger, sadness, depression, anxiety, fatigue, peer relationships, and pain) in pediatric burn patients. The BMS database is a national collection of burn patient outcomes from four centers in the United States. BMS outcomes collected were analyzed using multi-level, linear mixed effects regression modeling to examine associations between race/ethnicity and outcomes at discharge after index hospitalization, and 6- and 12-months post-injury. A total of 275 pediatric patients were included, of which 199 (72.3%) were Hispanic. After burn injury, of which the total body surface area was significantly associated with racial/ethnicity category (*p* < 0.01), minority patients more often reported higher levels of sadness, fatigue, and pain interference and lower levels of peer relationships compared to Non-Hispanic, White patients, although no significant differences existed. Black patients reported significantly increased sadness at six months (β = 9.31, *p* = 0.02) compared to discharge. Following burn injury, adult minority patients report significantly worse psychosocial outcomes than non-minority patients. However, these differences are less profound in pediatric populations. Further investigation is needed to understand why this change happens as individuals become adults.

## 1. Introduction

Burn survivors face complex physical and psychosocial challenges and often require multidisciplinary, longitudinal care. Following burn injury, patients regularly experience increased anxiety, depression, and posttraumatic symptoms compared to the general population [1]. Furthermore, approximately half of burn survivors encounter difficulties with social re-integration during their recovery [2,3]. Pediatric burn survivors are at high risk for adverse psychosocial outcomes due to their maturing emotional regulation, limited communication skills, and early stage of development [4]. Additionally, physical sequalae of burn injuries, such as scars, have a significant impact on psychosocial recovery in the domains of body image satisfaction and peer relationships [5]. The literature suggests evidence of long-term decreases in social functioning and increased social stigmatization in pediatric burn survivors [6] Further, pediatric patients often develop deficits in functional domains, such as language development and gross motor skills, even five years after injury [7,8].

In pediatric populations, racial and ethnic minority patients often face significant disparities in medical and psychosocial outcomes [9]. For example, after traumatic injury, African American and Hispanic patients experience higher mortality than White patients [10]. Minority pediatric patients are at higher risk for trauma exposure but have increased barriers to mental and medical healthcare [11,12,13]. Hispanic pediatric patients are at increased risk of intentional burn injury, inhalational injury, and larger burn sizes [14]. Regarding psychosocial outcomes, minority pediatric patients report increasing rates of depression, post-traumatic stress disorder, substance use disorder, and suicide [11,15].

Regarding burn injury, minority pediatric patients disproportionately represent the burn population [16,17]. Racial and ethnic minority patients are at higher risk for burn morbidity and mortality compared to non-minority patients [18]. These disparities are hypothesized to be influenced by inequities in socioeconomic status, healthcare status, and cultural factors [19]. In one study, 85% of parents of racial and ethnic minority patients report never receiving education regarding burn prevention or care [14]. Risk factors for burn injury, such as crowded households, simultaneous cooking and supervising children cultures, or even hair braiding practices, in minority patients have been identified, but investigations regarding post-burn outcomes in pediatric minority populations are lacking [7,14,18,20].

Little is known whether pediatric minority patients experience different psychosocial outcomes compared to White patients following burn injury, and whether these differences are comparable to adult populations. This study investigates seven domains of psychosocial outcomes, including levels of anger, sadness, depression, anxiety, fatigue, peer relationships, and pain, to determine whether disparities exist. This study utilizes the Burn Model System (BMS) database, established by the National Institute on Disability, Independent Living, and Rehabilitation Research (NIDILRR), to analyze these outcomes. We hypothesize, similar to that reported in adult minority populations, that significant disparities exist in post-burn psychosocial outcomes in minority pediatric patients. We aim to characterize these psychosocial outcomes to provide recommendations for optimal care in minority pediatric patients.

## 2. Methods

### 2.1. Study Design and Data Source

With study approval by our institutional review board, pertinent data from the BMS database was obtained. The BMS is a federally funded, multi-center program from four burn centers across the United States dedicated to research on long-term recovery after burn injury; the BMS program includes longitudinal data collection from pediatric and adult burn survivors. After enrollment, data is collected from patients at discharge through an in-person, mailed, or telephone survey. Participants are contacted 6-, 12-, and 24-months and every five years post-injury for data collection. This data is collected and managed by a BMS National Data and Statistics centers, with regular data quality tests.

For this study, data from the BMS program for this study was queried from 2005–2021. Inclusion criteria of participants consisted of: (1) patients must have had burn injury treated at one of the BMS centers, (2) patients consent to inclusion, (3) they meet the burn inclusion criteria. The burn inclusion criteria consisted of: (1) ≥10% TBSA, ≥65 years of age, and with burn surgery for wound closure, (2) ≥20% TBSA, 0–64 years of age, and with burn surgery for wound closure; (3) electrical high voltage/lightning with burn surgery for wound closure; (4) hand burn and/or face burn and/or feet burn with burn surgery for wound closure; or (5) patients meeting previous iterations of burn model system inclusion criteria. Patients must also have received acute care treatment in the BMS center from the time of the burn, including surgery for the closure of the burn wound, with closure occurring within 30 days of the burn injury. Exclusion criteria consisted of patients taken into law enforcement custody at admission or discharge from the BMS center. Further information regarding the BMS database’s data collection, burn centers, process, and inclusion criteria of participants can be found on the Burn Model System website (https://burndata.washington.edu/).

### 2.2. Variables of Interest

Demographics, such as age, gender, race, and ethnicity, were obtained from the BMS database. Race and ethnicity were re-coded as race/ethnicity, with three groups: Hispanic/Latino, Non-Hispanic White, and Black. Other variables included burn injury etiology and characteristics such as total surface burn area (TBSA), length of stay, and more. Patient reported outcomes regarding five domains, including levels of depression, anxiety, fatigue, peer relationships, and pain, were collected for three time-points (discharge, 6-, and 12-months post-injury) (Table S1). Patient reported outcomes for two domains, anger and sadness, were collected only at follow-up time-points (6- and 12-months post-injury). The total cohort of patients were divided into two groups as some outcomes were measured only in specific age ranges. The first cohort included patients between the ages of three and seven (Group A), and the second cohort consisted of patients between eight and 17 years old (Group B).

### 2.3. Quantitative Variables/Scale Outcomes

Patient reported outcomes in seven domains were collected utilizing questionnaires specific to pediatric or pediatric proxy participants. For Group A, data was collected from the National Institute of Health (NIH) Toolbox Anger and Sadness proxy surveys. The NIH Toolbox surveys are multidimensional sets of cognition, sensation, motor function, and emotional health measures that have been validated in pediatric populations [21]. Responses were provided by study participants’ parents or caregivers. For Group B, data was collected from the Patient-Reported Outcomes Measurement Information System (PROMIS) peer relationships, depressive symptoms, physical function, anxiety, fatigue, and pain interference surveys. The PROMIS measures collect physical, mental, and social health through validated surveys in burn populations [22,23]. Responses were provided directly from pediatric study participants.

### 2.4. Statistical Analyses

Patients baseline characteristics, clinical variables, and outcomes were reported as median (interquartile range) and were compared by the Kruskal–Wallis test for continuous variables. Frequency and percentage were reported for categorical variables and were compared using the Chi-square test or Fisher’s exact test. For longitudinal associations between race and outcomes, mixed effects linear regression models were utilized. Models used restricted maximum likelihood and empirical bayes method. Models used all available data points without data imputation.

Patient outcomes were measured at each time nested within each patient and then nested in four medical centers. Two random intercepts were included, with an autoregression structure for measurement along time and an exchange structure for medical centers. In the model, race/ethnicity, time, and the interaction of the two were included. Age, gender, TBSA, and number of operations were considered as potential confounders. Confounders were defined as changes in estimation of race/ethnicity over time over 10% with and without confounders. Confounder variables for the outcomes were also included in the final models. The analysis found that with and without multiple testing adjustments, the results were the same. Intra-class correlation analysis is provided in Supplementary Table S2. We reported the *p*-value directly without Bonferroni adjustment. The significance level was set as 0.05, 2-sided. All analyses were conducted using SAS 9.4 (SAS Institute Inc., Cary, NC, USA).

## 3. Results

### 3.1. Participants/Descriptive Data

A total of 275 pediatric patients were included in this study, with 111 patients in Group A and 164 patients in Group B. The average age of Group A was 4.6 years (standard deviation (SD): 1.14 years) and that of Group B was 11.5 years (SD: 2.9 years). Demographic data is presented in Table 1. Regarding Group A, there were 65 (58.6%) male and 46 (41.4%) female patients. Eighty-five (76.6%) patients were Hispanic, 18 (16.2%) were Non-Hispanic White, and eight (7.2%) were Black race and ethnicity. The average total body surface area (TBSA) of burns in this group was 39.2% (SD: 18.0%). The most common primary etiology of burn was fire/flame (*n* = 59, 53.2%) and scald (*n* = 45, 40.5%). In Group B, there were 116 (70.7%) male and 48 (29.3%) female patients. One-hundred-and-fourteen (69.5%) patients were Hispanic, 44 (26.8%) were Non-Hispanic White, and six (3.7%) were Black race and ethnicity. Average TBSA of burns was 40.2% (SD: 20.0%). The most common primary etiology of burns was fire/flame (*n* = 128, 78%), electrical (*n* = 17, 10.4%), and scald (*n* = 10, 6.1%).

In Group A, there was a significant association between TBSA (Non-Hispanic White: 20, Hispanic: 43, Black: 24.5, *p* < 0.01) and racial/ethnicity category. There was a significant association between race/ethnicity and location of burn injury on the torso (*p* = 0.01) and hands (*p* = 0.01). In Group B, there was a significant association between race/ethnicity and total physical function scores at discharge (Non-Hispanic White: 57.1, Hispanic: 45.5, Black: 39.5, *p* = 0.04) and six months (*p* = 0.01). There was no statistical significance at 12 months (*p* = 0.14). There was a significant association between TBSA (Non-Hispanic White: 26, Hispanic: 44, Black: 15, *p* < 0.01) and the racial/ethnicity category. No other statistically significant associations were found between race/ethnicity and survey follow-up. A significant association was found using Fisher’s exact test between race/ethnicity and the primary etiology of the injury (*p* = 0.03). Chi-square analysis revealed significant associations between race and ethnicity and the location of the burn injury on the torso (*p* < 0.01), perineum (*p* = 0.02), shoulder (*p* = 0.04), leg (*p* < 0.01), and foot (*p* = 0.03).

### 3.2. Outcome Data/Main Results

#### 3.2.1. Group A

##### Anger

Of the total 111 patients in Group A, 52 (46.8%) patients provided anger scores at six months and 66 (59.4%) patients at the 12-month follow-up. Reported anger scores were found to increase significantly with advancing age (β = 2.56, *p* = 0.02). Anger scores are presented in Table 2. Hispanic patients reported anger scores of 57.0 at six months and 57.5 at the 12-month follow-up. There were no statistical differences compared to Non-Hispanic White patients at the six-month (β = 0.49, *p* = 0.78) and 12-month follow-up (β = 2.13, *p* = 0.55). Black patients reported anger scores of 54.0 at six months and 51.0 at the 12-month follow-up. There were no statistical differences compared to Non-Hispanic White patients at six months (β = −2.55, *p* = 0.65) and 12 months (β = −4.33, *p* = 0.42) (Table 3).

##### Sadness

A total of 52 (46.8%) patients provided sadness scores at six months and 66 (59.5%) patients at the 12-month follow-up. Black patients reported significantly greater levels of sadness at 12 months after injury when compared to six months (β = 9.31, *p* = 0.02). Sadness scores are presented in Table 2. There were no statistical differences between Hispanic patients’ sadness scores compared to Non-Hispanic White patients at six months (β = 4.51, *p* = 0.21) and 12 months (β = 5.34, *p* = 0.10). Furthermore, there were no statistical differences between Black patients’ sadness scores compared to Non-Hispanic White patients at six months (β = −1.51, *p* = 0.77) and 12 months (β = 9.15, *p* = 0.06) (Table 3).

#### 3.2.2. Group B

##### Anxiety

In Group B, 89 (54.3%) patients reported anxiety scores at discharge, 75 (45.7%) patients at six months, and 75 (45.7%) patients at 12 months. Anxiety scores are presented in Table 4. There were no statistical differences between Hispanic and Non-Hispanic White patients’ anxiety scores at discharge (β = −0.39, *p* = 0.90), six months (β = 0.62, *p* = 0.85), and 12 months (β = 4.65, *p* = 0.19). There were no statistical differences between Black patients and Non-Hispanic White patients’ anxiety scores at discharge (β = 4.14, *p* = 0.54), at six months (β = −3.75, *p* = 0.68), and 12 months (β = −5.13, *p* = 0.51) (Table 5).

##### Depression

In Group B, 89 (54.2%) patients reported depression scores at discharge, 75 (45.7%) patients at six months, and 76 (46.3%) patients at 12 months. Depression scores are presented in Table 4. There were no statistically significant differences in depression scores between Hispanic and Non-Hispanic White patients at discharge (β = 1.33, *p* = 0.60), six months (β = 1.81, *p* = 0.51), and 12 months (β = 2.74, *p* = 0.35). There were no statistically significant differences between Black and Non-Hispanic White patients’ depression scores at discharge (β = −2.68, *p* = 0.65), six months (β = −1.37, *p* = 0.85), and 12 months (β = −3.90, *p* = 0.56) (Table 5).

##### Fatigue

In Group B, 87 (53.0%) patients reported fatigue scores at discharge, 74 (45.1%) patients at six months, and 74 (45.1%) patients at 12 months. Reported fatigue scores were found to be significantly higher with increased age (β = 0.79, *p* = 0.02) and number of operations (β = 0.95, *p* < 0.01). Fatigue scores are presented in Table 4. There were no statistically significant differences between Hispanic and Non-Hispanic White patients’ fatigue scores at discharge (β = 4.18, *p* = 0.18), six months (β = 5.56, *p* = 0.10), and 12 months (β = 4.75, *p* = 0.19). There were no statistically significant differences between Black and Non-Hispanic White patients’ fatigue scores at discharge (β = 1.87, *p* = 0.79), six months (β = 3.14, *p* = 0.74), and 12 months (β = −2.02, *p* = 0.80) (Table 5).

##### Peer Relationships

Regarding peer relationships, 86 (52.4%) patients reported scores at discharge, 73 (44.5%) patients at six months, and 76 (46.3%) patients at 12 months. Peer relationship scores are presented in Table 4. There were no statistically significant differences between Hispanic and Non-Hispanic White patients’ peer relationship scores at discharge (β = −1.32, *p* = 0.62), six months (β = 1.33, *p* = 0.65), and 12 months (β =−4.15, *p* = 0.17). There were no statistically significant differences between Black and Non-Hispanic White patients’ peer relationship scores at discharge (β = −3.20, *p* = 0.58), six months (β = −2.03, *p* = 0.79) and 12 months (β = 3.88, *p* = 0.56) (Table 5).

##### Pain Interference

In Group B, 87 (53.0%) patients reported pain interference scores at discharge, 75 (45.7%) patients at six months, and 76 (46.3%) patients at 12 months. In the total cohort, reported pain interference was significantly higher with increased number of operations (β = 0.89, *p* < 0.01). Pain interference scores are presented in Table 4. There were no statistically significant differences between Hispanic and Non-Hispanic White patients’ pain interference scores at discharge (β = 3.36, *p* = 0.11), six months (β = 2.32, *p* = 0.30), and 12 months (β = 3.99, *p* = 0.10). There were no statistically significant differences between Black and Non-Hispanic White patients’ pain interference scores at discharge (β = 2.94, *p* = 0.54), six months (β = 8.32, *p* = 0.17), and 12 months (β = 1.42, *p* = 0.80) (Table 5).

## 4. Discussion

This study investigated seven domains to determine whether psychosocial disparities found in adult minority populations also exist in pediatric minority populations after burn injury. In this study, we report worse psychosocial outcomes in minority pediatric populations, although differences were not significant. Currently, the literature shows that significant disparities in adult minority populations that exist in outcomes such as community integration, higher overall pain severity, increased post-traumatic stress symptomology, and greater dissatisfaction with appearance are well known, but less is known regarding pediatric populations [14,24,25]. Furthermore, in racial and ethnic minority patients, increased levels of depression, anxiety, and social functioning are reported [26,27]. These racial and ethnic minority burn survivors may face stigma that compounds to the added burden such as individual or structural levels of discrimination that certainly impart psychosocial outcomes after burn recovery [28].

In the literature detailing outcomes after other traumatic injuries, psychosocial disparities affecting racial and ethnic minorities persist. After traumatic brain injury, Black patients had increased levels of depression, posttraumatic stress symptoms, and anxiety and lower levels of social integration and cognitive function scores compared to White patients [29,30]. Minority patients were more likely to have decreased community integration, to report worse social support, more depressive symptoms, and less confidence in physical function after traumatic injury [30]. In traumatic spinal cord injury, minority patients were significantly more likely to report depressive symptoms during recovery than non-minority patients [31]. Regarding pediatric populations, Hispanic trauma patients were more likely to report lower scores on quality of life and adaptive behavior assessments after injury compared to Non-Hispanic, White patients [32]. However, research is limited regarding psychosocial outcomes after traumatic injury, including burn injury, in pediatric minority patients.

In our study, although no significant differences were found in adverse psychosocial outcomes, Hispanic pediatric patients experienced higher levels of sadness, depression, fatigue, and pain interference than White pediatric patients at all time points. Further, Black pediatric patients reported higher levels of pain interference than White pediatric patients at all time points. Our findings warrant further investigation to delineate psychosocial outcomes in minority pediatric burn populations to provide the best care. It is unclear whether or not differences in psychosocial outcomes exist for minority pediatric patients compared to White patients, high rates of child anxiety, behavioral problems, and depressive symptoms exist after burn injuries [33,34]. During short-term burn recovery, adverse sequalae, such as increased anxiety and social withdrawal, are common in all populations [35]. Regarding burn injury, minority pediatric patients have higher TBSA, increased risk for injury, and worse functional outcomes compared to White patients [36,37]. Additionally, minority pediatric patients are at higher risk for intentional burn injuries compared to White patients [37]. Although in our study, Non-Hispanic White patients were utilized as reference, further research is necessary to determine whether significant differences in post-burn psychosocial recovery exist within racial and minority groups. Differences have been reported regarding psychosocial and socioeconomic factors that may influence post-burn outcomes. For example, a study investigating psychosocial differences between Hispanic and Black patients found that Black mothers often reported more social support and higher self-esteem than Hispanic mothers [38]. However, no studies investigating psychosocial factors influencing pediatric populations after burns exist. A better understanding of these differences may help provide guidance to better support these patients during their recovery.

Currently, outpatient modalities exist to provide support to minority pediatric burn survivors during burn recovery. Participation in peer burn support programs demonstrate improvements to psychosocial outcomes including greater social integration and reductions in post-traumatic stress and anxiety [39]. However, these peer support programs should be inclusive to patients with limited English proficiency and readily accessible to be efficacious. Furthermore, these programs should address barriers commonly cited by minority patients, such as lack of time, low awareness of programs, and distance to participate in programs [40]. Similarly, burn camps have been reported to improve self-esteem and social integration in participants. However, even within these camps, racial and ethnic minority participants report lower improvements to self-esteem compared to White participants [41,42]. Potentially delivering culturally competent care by providing multi-lingual options, racial and ethnic patient-provider concordance, and promoting understanding of minority experiences may improve psychosocial outcomes of minority pediatric patients at institutional programs such as burn camps.

Some limitations of this study include those associated with utilizing a single database for data collection. Missing data points from significant follow-up attrition may have affected statistical significance and introduced a potential for selection bias. There was a fraction of participants not completing follow-up after discharge, which represents a significant limitation that has clinical implications. Future studies should seek to determine factors impacting poor follow-up in certain minority populations and address them. Furthermore, the BMS data set did not include patient pre-burn characteristics, such as personality, socioeconomic status, faith, or family support, which may serve as potential confounders. Further research should utilize pre-burn patient characteristics to further interpret post-injury outcomes [43,44] Additionally, outcome measures in the BMS dataset utilized different assessment tools for our populations, which prevented statistical analysis between our two pediatric populations. Another limitation included the patient sample as the majority of patients were Hispanic, with under-representation of other minority patients. Potential geographic confounders regarding the distribution of the survey and participants may influence the results presented in this study. Furthermore, for Group A, parental proxy questionnaires were utilized to gather levels of anger and sadness outcomes in pediatric patients. As racial and ethnic minority parents of pediatric burn patients are more likely to experience increased posttraumatic stress, depression, and guilt, proxy questionnaires may include some level of confounding bias [45].

Another potential limitation may be the “response shift bias”, as self-reported outcomes were collected at discharge after treatment for burn injury and participants’ frame of reference regarding outcomes may have changed over the study period. Lastly, the criteria for inclusion into the BMS database, as provided in the Methods, include patients with more serious burn injuries, which may reduce generalizability to pediatric patients with more minor burn injuries. Future studies should seek to utilize a larger cohort of pediatric minority patients and report on outcomes greater than one year from burn injury. In addition, studies should seek to prospectively investigate social determinants of health in both pediatric and adult minority patient populations to determine and address specific areas contributing to psychosocial disparities following burn injury. This investigation should also seek to determine differences in psychosocial recovery within different racial and ethnic groups and describe factors that contribute to variations in recovery.

## 5. Conclusions

We found, in our cohort, that minority pediatric patients experienced larger burn injuries and were younger than non-minority patients. However, significant differences were not reported regarding psychosocial outcomes in minority patients compared to non-minority patients. From previous studies, adult minority patients have worse psychosocial outcomes and minority pediatric patients often experience worse psychosocial outcomes in other traumatic injuries. Further investigation is needed to understand why this change happens as individuals become adults.

## Figures and Tables

**Table 1:** Patient demographic data.

Characteristics	Group A	Non- Hispanic White (*n* = 18)	Black (*n* = 8)	Hispanic (*n* = 85)	Group B	Non- Hispanic White (*n* = 44)	Black (*n* = 6)	Hispanic (*n* = 114)
Age, mean years (SD)	4.7 (1.1)	5.1 (1.1)	4.4 (1.0)	4.6 (1.2)	11.5 (2.9)	12.0 (2.8)	11.5 (2.7)	11.4 (2.9)
Average Total Body Surface Area (TBSA), %	39.2% (18.0)	20.0% (12.5)	27.9% (18.5)	44.3% (15.8)	40.2% (20.0)	28.9% (22.2)	25.5% (24.8)	45.4% (16.5)
Sex, *n* (%)								
Male	65 (58.6%)	9 (50%)	4 (50%)	52 (61.2%)	116 (70.7%)	34 (77.3%)	3 (50%)	116 (70.7%)
Female	46 (41.4%)	9 (50%)	4 (50%)	33 (38.8%)	48 (29.3%)	10 (22.7%)	3 (50%)	48 (28.3%)
Primary Etiology of Injury, *n* (%)								
Flame	59 (53.2%)	14 (77.8%)	2 (25%)	43 (50.6%)	128 (78.0%)	35 (79.5%)	4 (66.7%)	89 (78.1%)
Scald	45 (40.5%)	4 (22.2%)	5 (62.5%)	36 (42.4%)	10 (6.1%)	3 (6.8%)	0 (0%)	7 (6.1%)
Grease	3 (2.7%)	0 (0%)	0 (0%)	3 (3.5%)	3.1 (1.8%)	1 (2.3%)	1 (16.7%)	1 (0.9%)

**Table 2:** Group A reported sadness and anger scores.

Race and Ethnicity	Time	Total Sadness Score (*n*)	95% Confidence Interval (*n*)	Total Anger Score (*n*)	95% Confidence Interval (*n*)
Non-Hispanic White (*n*= 18)					
	Discharge	NA	NA	NA	NA
	6 Months	51.20 (10)	44.87, 57.52	56.53 (10)	49.63, 63.43
	12 Months	49.84 (10)	44.16, 55.53	55.32 (10)	49.08, 61.56
Hispanic/Latino Hispanic (*n* = 85)					
	Discharge	NA	NA	NA	NA
	6 Months	55.70 (35)	52.36, 59.04	56.97 (35)	53.34, 60.59
	12 Months	55.18 (35)	52.11, 58.25	57.45 (35)	54.11, 60.80
Black (*n* = 8)					
	Discharge	NA	NA	NA	NA
	6 Months	49.69 (7)	41.87, 57.50	53.98 (7)	45.43, 62.53
	12 Months	58.99 (7)	51.13, 66.85	51.00 (7)	42.41, 59.58

Group A reported sadness and anger scores at three time points with racial/ethnic category breakdown.

**Table 3:** Sadness and anger scores in minorities compared to Non-Hispanic White patients.

Time Points	Non-Hispanic White (Reference)	Hispanic/Latino *	Black *
Sadness			
Discharge	0.00	NA	NA
6 Months	0.00	4.51 (3.59, *p* = 0.21)	−1.51 (5.05, *p* = 0.77)
12 Months	0.00	5.34 (3.24, *p* = 0.10)	9.15 (4.87, *p* = 0.06)
Anger			
Discharge	0.00	NA	NA
6 Months	0.00	0.49 (3.91, *p* = 0.78)	−2.55 (5.54, *p* = 0.65)
12 months	0.00	2.13 (3.56, *p* = 0.55)	−4.33 (5.35, *p* = 0.42)

*: Beta Estimate, (Standard Error, *p*-Value). Group A sadness and anger scores in racial/ethnic minority patients with Non-Hispanic White patients as reference.

**Table 4:** Group B reported psychosocial outcome scores.

Race and Ethnicity	Time	Total Anxiety Score (95% Confidence Interval (CI)), (*n*)	Total Depression Score (95% CI), (*n*)	Total Fatigue Score (95% CI), (*n*)	Total Peer Relationship Score (95% CI), (*n*)	Total Pain Interference Score (95% CI), (*n*)
Non-Hispanic White (*n* = 44)						
	Discharge	45.10 (40.26, 49.94), (26)	45.99 (41.75, 50.23), (26)	46.19 (41.12, 51.27), (26)	53.71 (49.30, 58.12), (25)	41.09 (37.58, 44.60), (25)
	6 Months	44.42 (39.04, 49.79), (21)	46.30 (41.73, 50.88), (21)	44.50 (38.90, 50.10), (21)	52.21 (47.42, 57.00), (20)	41.84 (38.10, 45.59), (21)
	12 Months	41.24 (35.10, 47.38), (16)	43.76 (38.64, 48.88), (16)	41.82 (35.49, 48.15), (16)	55.47 (50.23, 60.70), (16)	38.18 (34.01, 42.35), (16)
Hispanic/Latino (*n* = 114)						
	Discharge	44.71 (41.50, 47.92), (59)	47.32 (44.55, 50.10), (59)	50.37 (46.97, 53.77), (57)	52.39 (49.00, 55.78), (57)	44.45 (42.18, 46.72), (58)
	6 Months	45.04 (41.62, 48.45), (52)	48.11 (45.21, 51.01), (52)	50.06 (46.49, 53.63), (51)	53.54 (50.05, 57.02), (51)	44.17 (41.80, 46.53), (52)
	12 Months	45.89 (42.60, 49.18), (56)	46.50 (43.68, 49.32), (57)	46.57 (43.11, 50.03), (55)	51.32 (47.95, 54.68), (57)	42.16 (39.87, 44.46), (57)
Black (*n* = 6)						
	Discharge	40.96 (28.60, 53.32), (4)	43.31 (32.40, 54.21), (4)	48.06 (35.08, 61.05), (4)	50.52 (39.72, 61.32), (4)	44.04 (35.13, 52.94), (4)
	6 Months	40.67 (23.34, 58.00), (2)	44.93 (30.94, 58.92), (2)	47.64 (29.85, 65.44), (2)	50.18 (36.03, 64.33), (2)	50.16 (38.74, 61.58), (2)
	12 Months	36.11 (21.88, 50.34), (3)	39.86 (27.62, 52.09), (3)	39.80 (24.94, 54.65), (3)	59.35 (47.26, 71.44), (3)	39.60 (29.61, 49.59), (3)

Group B reported psychosocial outcome scores for anxiety, depression, fatigue, peer relationship, and pain interference at three time points with racial/ethnic category breakdown.

**Table 5:** Reported psychosocial outcomes in Group B.

Time Points	Non-Hispanic White (Reference)	Hispanic/Latino *	Black *
Anxiety			
Discharge	0.00	−0.39 (2.93, *p* = 0.90)	−4.14 (6.69, *p* = 0.54)
6 Months	0.00	0.62 (3.21, *p* = 0.85)	−3.75 (9.15, *p* = 0.68)
12 Months	0.00	4.65 (3.51, *p* = 0.19)	−5.13 (7.82, *p* = 0.51)
Depression			
Discharge	0.00	0.43 (3.91, *p* = 0.91)	−2.55 (5.54, *p* = 0.65)
6 Months	0.00	2.13 (3.56, *p* = 0.55)	−4.33 (5.35, *p* = 0.42)
12 Months	0.00	−3.47 (4.34, *p* = 0.43)	−8.18 (6.14, *p* = 0.19)
Fatigue			
Discharge	0.00	4.18 (3.07, *p* = 0.18)	1.87 (7.02, *p* = 0.79)
6 Months	0.00	5.56 (3.35, *p* = 0.10)	3.14 (9.41, *p* = 0.74)
12 Months	0.00	4.75 (3.63, *p* = 0.19)	−2.02 (8.14, *p* = 0.80)
Peer Relationships			
Discharge	0.00	−1.32 (2.69, *p* = 0.62)	−3.20 (5.80, *p* = 0.58)
6 Months	0.00	1.33 (2.88, *p* = 0.65)	−2.03 (7.46, *p* = 0.79)
12 Months	0.00	−4.15 (3.01, *p* = 0.17)	3.88 (6.58, *p* = 0.56)
Pain Interference			
Discharge	0.00	3.36 (2.11, *p* = 0.11)	2.94 (4.83, *p* = 0.54)
6 Months	0.00	2.32 (2.23, *p* = 0.30)	8.32 (6.07, *p* = 0.17)
12 Months	0.00	3.99 (2.40, *p* = 0.10)	1.42 (5.47, *p* = 0.80)

*: Beta Estimate, (Standard Error, *p*-Value). Group B psychosocial outcomes in racial/ethnic minority patients with Non-Hispanic White as a reference.

## Data Availability

The data presented in this study are available on request from the corresponding author. The data are not publicly available due to protected database source.

## Supplementary Materials

The following supporting information can be downloaded at: https://www.mdpi.com/article/10.3390/ebj4020015/s1, Table S1: Variables of Interest; Table S2: Intra-Class Correlation Analysis.
