# Supplementary material for: Post-Burn Psychosocial Outcomes in Pediatric Minority Patients in the United States: An Observational Cohort Burn Model System Study
Source: Eur Burn J. 2023 Apr 3;4(2):173–83. doi: 10.3390/ebj4020015 (PMC10290777; doi:10.3390/ebj4020015)
Supplement: Supplementary file 1 [file ebj-04-00015-s001.zip › ebj-2233132-supplementary.pdf]

Supplementary Tables

Table S1. Variables of Interest.

Category	Variables of Interest	Collection Methodology
Patient Demographics	Age, gender, race/ethnicity, pre-burn comorbidities	BMS center staff person and original coder.
Burn Injury Characteristics	Burn size, etiology, location of injury	BMS center staff person and original coder.
In-patient Hospitalization	Length of stay	BMS center staff person and original coder.
Patient Reported Outcomes	Anger, sadness, depression, anxiety, fatigue, peer relationships, pain	Patient-Reported Outcomes Measurement Information System (PROMIS) Pediatric and Parent Proxy Profile Instruments, National Institute of Health Toolbox.

Table S2. Intra-Class Correlation Analysis.

	**Intra-Class Correlation (Patient vs. Time Point)**	**Intra-Class Correlation (Site Specific Patients Vs. Time Point) ***
Anger	63.15%	63.41%
Sadness	61.45%	61.45%
Anxiety	12.76%	12.77%
Depression	56.59%	56.60%
Fatigue	32.75%	32.75%
Peer Relationships	47.60%	47.61%
Pain Interference	57.29%	57.30%

* Intra-Class Correlation of scores at different time points within same patient within specific site.
